# Interferon-Gamma (IFN-γ)-Mediated Retinal Ganglion Cell Death in Human Tyrosinase T Cell Receptor Transgenic Mouse

**DOI:** 10.1371/journal.pone.0089392

**Published:** 2014-02-28

**Authors:** Shahid Husain, Yasir Abdul, Christine Webster, Shilpak Chatterjee, Pravin Kesarwani, Shikhar Mehrotra

**Affiliations:** 1 Hewitt Laboratory of the Ola B. Williams Glaucoma Center, Department of Ophthalmology, Storm Eye Institute, Medical University of South Carolina, Charleston, South Carolina, United States of America; 2 Department of Surgery, Hollings Cancer Center, Medical University of South Carolina, Charleston, South Carolina, United States of America; University of Colorado, Denver, United States of America

## Abstract

We have recently demonstrated the characterization of human tyrosinase TCR bearing h3T-A2 transgenic mouse model, which exhibits spontaneous autoimmune vitiligo and retinal dysfunction. The purpose of current study was to determine the role of T cells and IFN-γ in retina dysfunction and retinal ganglion cell (RGC) death using this model. RGC function was measured by pattern electroretinograms (ERGs) in response to contrast reversal of patterned visual stimuli. RGCs were visualized by fluorogold retrograde-labeling. Expression of CD3, IFN-γ, GFAP, and caspases was measured by immunohistochemistry and Western blotting. All functional and structural changes were measured in 12-month-old h3T-A2 mice and compared with age-matched HLA-A2 wild-type mice. Both pattern-ERGs (42%, p = 0.03) and RGC numbers (37%, p = 0.0001) were reduced in h3T-A2 mice when compared with wild-type mice. The level of CD3 expression was increased in h3T-A2 mice (h3T-A2: 174±27% vs. HLA-A2: 100%; p = 0.04). The levels of effector cytokine IFN-γ were also increased significantly in h3T-A2 mice (h3T-A2: 189±11% vs. HLA-A2: 100%; p = 0.023). Both CD3 and IFN-γ immunostaining were increased in nerve fiber (NF) and RGC layers of h3T-A2 mice. In addition, we have seen a robust increase in GFAP staining in h3T-A2 mice (mainly localized to NF layer), which was substantially reduced in IFN-γ^ (-/-)^ knockout h3T-A2 mice. We also have seen an up-regulation of caspase-3 and -9 in h3T-A2 mice. Based on our data we conclude that h3T-A2 transgenic mice exhibit visual defects that are mostly associated with the inner retinal layers and RGC function. This novel h3T-A2 transgenic mouse model provides opportunity to understand RGC pathology and test neuroprotective strategies to rescue RGCs.

## Introduction

Retinal ganglion cell (RGC) death is a common event in numerous retinopathies and optic neuropathies including glaucoma. Several theories have been proposed for RGC death; however; the pathological process for RGC death has remained poorly defined. Numerous factors have been identified that contribute directly or indirectly in the RGC death process. These factors include: biomechanical stress (e.g., elevated intraocular pressure), oxidative stress, neuroinflammation, alteration in neurotrophic signaling, excitotoxicity, protein misfolding, glial activation, mitochondrial dysfunction, hypoxia/ischemia, genetic mutation, and auto-immunity [1]–[5]. RGCs are highly vulnerable in numerous retinopathies [6]–[10], but there is no effective therapy to prevent/delay RGC death in such retinopathies where RGCs are at high risk. Thus, a better understanding of the complex network of RGC death mediators is needed.

Although eyes are arguably the most vulnerable, but also the most “immune privileged” organ; paradoxically, eyes remain subject to destructive autoimmunity that may result after inflammatory reaction triggered by environmental (microbial, stress) and autologous (tissue damage) “danger” signals [11]. The healthy eye is sequestered behind an efficient blood-retina barrier to the entry of unwanted molecules, while remaining under a profoundly immunosuppressive microenvironment [12], [13]. However, under certain pathological conditions these barriers and the protective microenvironment can be compromised and certain non-tolerant T cells can infiltrate the retina. The discovery of activated T cells and macrophages in the brain parenchyma of neurodegenerative disease confirms the involvement of a cellular immune response within the central nervous system [14]–[16]. While some studies have also suggested that immune cells (particularly T cells) may play key roles in RGC death, a precise role for T cells in retinopathies remains poorly defined. Our data herein provides evidence for a pathological role for T cells and a cytokine (interferon-gamma, IFN-γ) in RGC death in a spontaneously depigmentating T cell receptor (TCR) transgenic mouse model h3T-A2 [17], [18]. Our data suggests that the activation of tyrosinase epitope YMDGTMSQV reactive TCR transgenic T cells triggers the release of inflammatory cytokines and thereby initiates neurodegenerative responses. The availability of this unique mouse model provided us an opportunity to understand the cellular events in which T cells and IFN-γ play crucial roles in deciding the fate of RGCs.

## Materials and Methods

### Animals

C57BL/6 mice, HLA-A2, and h3T-A2 mice (11–13 months-of-age; 30–40 grams) were used in this study. Mice were kept under a cycle of 12-hours light and 12-hours dark for all the studies. Animal handling was performed in accordance with the Association for Research in Vision and Ophthalmology Statement for the Use of Animals in Ophthalmic and Vision Research; and the study protocol was approved by the Animal Care and Use Committee at the Medical University of South Carolina. The development of h3T-A2 mice has been described previously [17]. Effector-molecule deficient h3T-A2 mice were obtained by crossing h3T-A2 mice with TNF-α^-/-^ (Stock No. 003008), IFN-γ^-/-^ (Stock No. 002287) and perforin^-/-^ mice (Stock No. 002407). All the deficient mice were obtained from Jackson Laboratory (Jackson Laboratory, Bar Harbor, ME). Animals were maintained in pathogen-free facilities and under the approved procedures of the Institutional Animal Care and Use Committee.

### Pattern-electroretinogram (Pattern-ERG) recordings

Mice were anesthetized with ketamine (80 mg/kg) and xylazine (10 mg/kg) and body temperature was maintained at 37°C with a heating pad. The Pattern-ERG electrode was placed on the corneal surface by means of a micromanipulator and positioned in such a way as to encircle the undilated pupil without limiting the field of view. A small drop of saline was applied to keep the cornea and lens moist during each recording. A visual stimulus generated by black and white alternating contrast reversing-bars (mean luminance, 50 cd/m^2^; spatial frequency, 0.033 cycles/deg; contrast, 99%; and temporal frequency, 1 Hz) was aligned with the projection of the undilated pupil at an 11 cm distance using the UTAS-2000 (LKC Technologies) visual diagnostic system. Each Pattern-ERG was an average of 300 sweeps at an interval of 1 second. For the Pattern-ERG amplitudes, measurements were made between a peak and adjacent trough of the waveform as described earlier [19], [20].

### Retrograde-labeling of retinal ganglion cells

Mice were deeply anesthetized with ketamine (80 mg/kg), xylazine (10 mg/kg), and body temperature was maintained at 37°C with a heating pad. Retrograde-labeling of RGCs was performed as described earlier [19], [20]. Briefly, 2 µL of a 5% solution of fluorogold in PBS was injected into the superior colliculus of mice immobilized in a stereotaxic apparatus. Using a small drill, a 1/8″ hole was made in the skull 2.92 mm from bregma and 0.5 mm from lambda. After making this hole in the skull, a Hamilton syringe was filled with fluorogold and the syringe needle gently inserted at the hole and going down 2 mm, whereupon the fluorogold was injected. The needle was left in the brain for 30–60 second and then slowly removed. The skull hole was filled with bone wax 903 (Lukens Cat # 2007-05). Seven days post injection, animals were euthanized and their eyes were enucleated and fixed in 4% paraformaldehyde (PFA) for 24 hour at 4°C. After rinsing with PBS, each retina was detached from the eyecup and prepared as a flatmount with the vitreous-side up. Each retina was divided into four quadrants, and each quadrant was further divided in two regions (inner and peripheral retina). RGCs were counted in exactly the same fashion in wild-type and h3T-A2 transgenic mice. RGCs were counted and averaged per 8 microscopic fields of identical size (150 µm^2^; 20× magnification) per retina by using Image J software (NIH, Bethesda, MD). The automated RGC numbers generated by Image J software were comparable when RGCs were counted manually by two operators in a masked fashion.

### Immunohistochemistry

Eyes were enucleated and immunohistochemistry was performed as described previously [20], [21]. After removing the anterior segment of the eye and the lens, eyes were fixed in 4% PFA for 4 hours, then cryoprotected in 25% sucrose solution overnight at 4°C. The eyecups were washed in ice-cold phosphate-buffered saline and frozen in OCT embedding medium over dry ice. Eyes were either stored at −20°C or cryosections were cut. Cryosections were cut at −20°C, fixed in cold methanol for 10 minutes, and rinsed in 1× Tris-buffered saline (TBS), pH 7.5. Tissues were permeabilized with 0.2% Triton-X-100 in TBS and washed again with TBS. Tissues were then blocked with 5% bovine serum albumin (BSA) in TBS for 1 hour at room temperature, followed by incubation with primary antibodies (e.g., anti-CD3 antibody, 1∶100 dilution; anti-NeuN antibody, 1∶100; anti-IFN-γ, 1∶100 dilution; anti-GFAP, 1∶200 dilution; anti-caspase-3, 1∶100 dilution; or 0.5% BSA) for overnight at 4°C. Cryosections were then washed with TBS and incubated with fluorescein-conjugated secondary antibody (anti-mouse IgG, 1∶400; DyLight™ 488 and anti-rabbit IgG, 1∶600 rhodamine (Jackson Immuno Research Laboratories, Inc., West Grove, PA) at room temperature for 1 hour. Negative control slides were incubated with 0.5% BSA in place of the primary antibody. The sections were observed under a bright-field microscope equipped with epifluorescence, and digitized images were captured by a digital camera.

### Western blotting

Equivalent amounts of retina extracts (15–30 µg protein/lane) were subjected to 10% SDS-PAGE, proteins separated, and proteins transferred to nitrocellulose membranes as described earlier [22]. The membranes were blocked with 5% non-fat dry milk followed by incubation for 12 h at 4°C with appropriate primary antibodies (e.g., anti-CD3, anti-IFN-γ, anti-caspase-3, and anti-caspase-9 at 1∶1000 dilution or anti-β-Actin at 1∶3000 dilution). After washing, membranes were incubated for 1 h at 20°C with appropriate secondary antibodies (HRP-conjugated; dilution 1∶3000). Pre-stained molecular weight and magic markers were run in parallel to identify the molecular weight of proteins of interest. For chemiluminescent detection, the membranes were treated with enhanced chemiluminescent reagent, and the signal was monitored using a Biorad Versadoc imaging system (Biorad, Hercules, CA).

### ELISA of IFN-γ and TNF-α

Splenocytes from h3T-A2 mice or effector molecules deficient (h3T-A2IFN-γ^-/-^, h3T-A2TNF-α^-/-^ and h3TA2Perforin^-/-^) mice cells were plated into U-bottomed 96-well plates (1×10^5^ cells per well) with IMDM medium. Plated cells were co-cultured overnight with either cognate antigen h-Tyr (1 µg/ml) or control MART-1 (1 µg/ml) antigen, pulsed with surrogate antigen presenting T2-A2 cells (Effector: T2-A2 cells ratio-10:1). Concentrations of mouse IFN-γ and TNF-α were measured in supernatants at overnight with an IFN-γ ELISA kit (R&D Systems, Minneapolis, MN) and mouse TNF-α ELISA kit (eBioscience, San Diego, CA).

### Statistical analysis

Statistical comparisons were made using the Student *t* test for paired data or ANOVA using the Bonferroni post-test for multiple comparisons (GraphPad Software, Inc., San Diego, CA). *p*≤0.05 was considered significant.

## Results

### Morphological and ocular functional deficits in h3T-A2 transgenic mice

**Figure 1:**
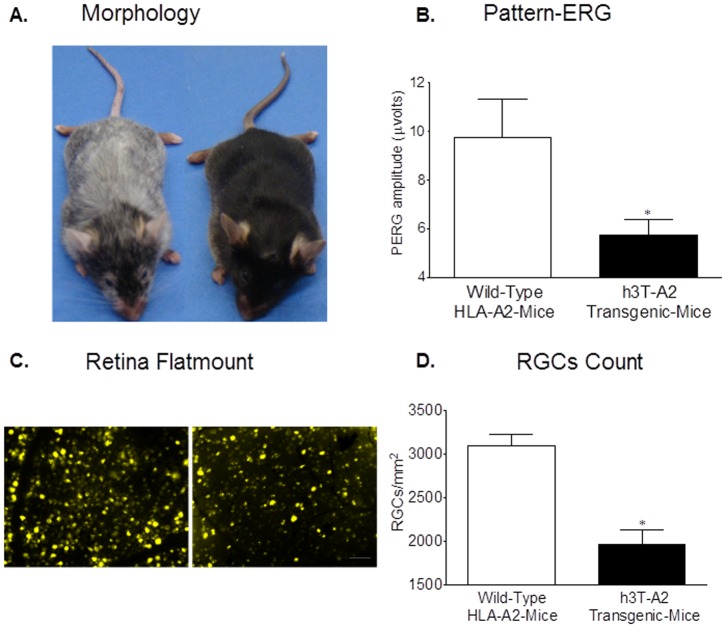
(**A**) h3T-A2 mouse with spontaneous vitiligo. Double-transgenic h3T-A2 mouse along with non-transgenic littermate at 18 months-of-age. Development of spontaneous progressive vitiligo in double-transgenic mouse on extreme left is noticeable after five weeks-of-age. Age-matched h3T-A2 mouse shows de-pigmentation. (**B**) Pattern-ERG recording in 12-month-old wild-type HLA-A2 and h3T-A2 mice. Each waveform is a mean of 300 individual waveforms taken at an interval of 1 second for each data point. Data are mean ±SE; *p<0.05; n = 6–7. (**C**) Fluorescence micrographs of flat-mounted retinas depicting Fluorogold-labeled retina ganglion cells (RGCs) in 12-month-old wild-type HLA-A2 and h3T-A2 mice. Briefly, 2 µL of a 5% solution of fluorogold was injected into the superior colliculus of anesthetized animals. Seven days post injection, animals were euthanized and retinas were prepared as flat-mounts, vitreous-side facing up. Fluorescent RGCs were visualized under Zeiss microscopy. (**D**) RGCs were counted in 8 microscopic fields of identical size (150 µm^2^ area) for each retina using Image J software. *p<0.05; n = 6 for each group.

The development and basic characterization of the h3T-A2 mice has been described recently [18]. The phenotype of the h3T-A2 and control wild-type mouse is shown in Figure 1A. Note the marked spontaneous areas of depigmentation or vitiligo among the double-transgenic h3T-A2 mice at 6–8 weeks-of-age. The progressive vitiligo observed in the h3T-A2 mice from two weeks onward is due to melanocyte destruction in the skin [18]. We have evaluated the h3T-A2 transgenic mouse model for retinal ganglion cell (RGC) function by measuring the pattern-electroretinogram (Pattern-ERG) and RGC death by retina flat-mounts in hopes that the h3T-A2 model may offer pathological resemblance to the ocular disease (e.g., glaucoma) in which RGCs are dying primarily during the progression of the disease. Studies in human, primates, and rodents have shown that pattern-ERG is a measure of RGC function [19], [23]. Pattern-ERG is obtained in response to contrast reversal of patterned visual stimuli (gratings, checkerboards), rather than uniform flashes of light as described in our recently published manuscripts [19], [20]. As shown in Figure 1B, pattern-ERGs were reduced by 42% (*p* = 0.03; n = 6–7) in 12-month-old h3T-A2 mice when compared with age-matched HLA-A2 mice. Additionally, the number of RGCs was reduced by 37% (*p*<0.05; n = 6) in h3T-A2 mice when compared with age-matched HLA-A2 mice (Fig. 1C–D).

**Figure 2:**
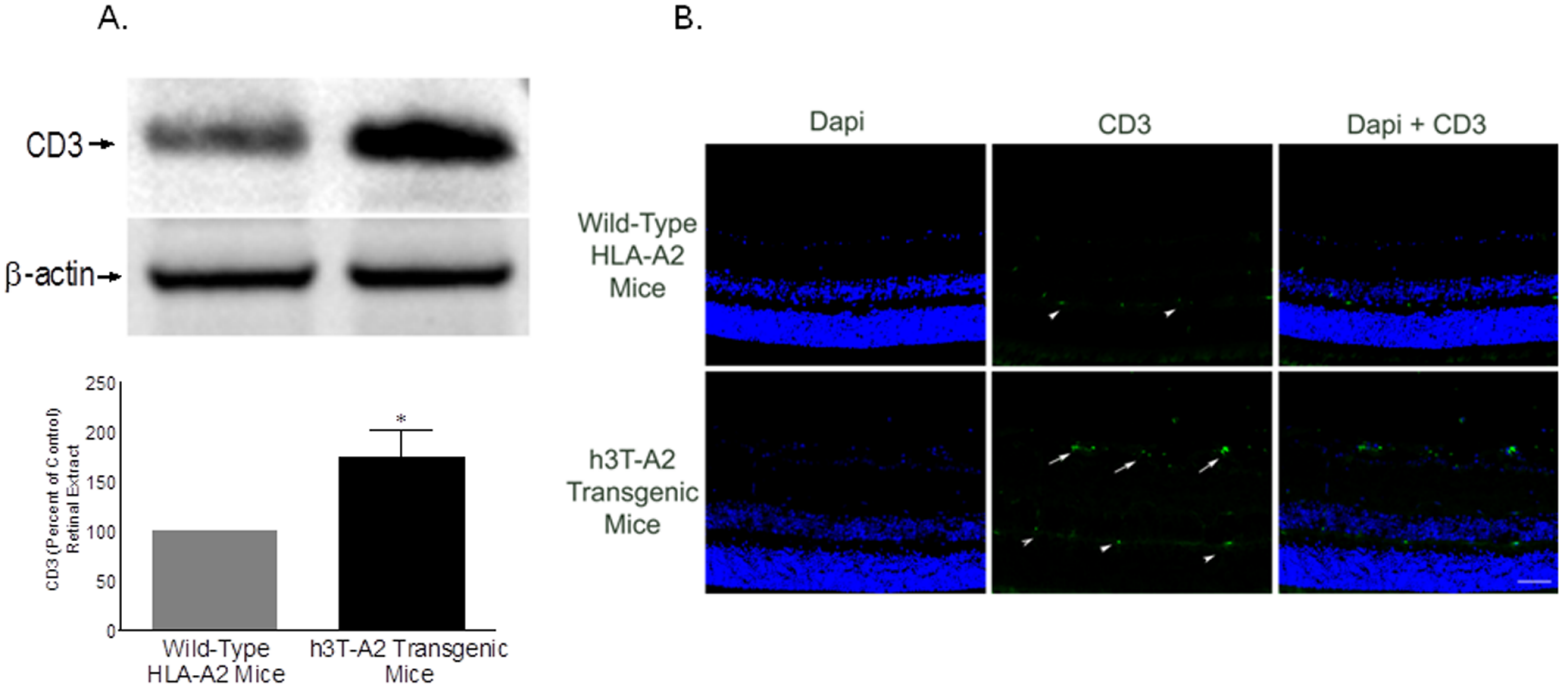
Detection of CD3+ in the retina of 12-month-old wild-type HLA-A2 and h3T-A2 mice by Western blotting (A) and immunohistochemistry (B). Data are expressed as mean ±SE. *p<0.05; n = 6 for each group. Bar size is 20 µm. Arrows indicate CD3 staining; arrowheads indicate non-selective staining, as also observed in the negative control (not shown).

We also have demonstrated earlier that h3T-A2 transgenic T cells infiltrated into the retina of h3T-A2 mice as determined by *in situ* hybridization [18], suggesting a neurodestructive role for T cells in the retina. To confirm the protein levels of infiltrated CD3^+^ T cells within the retina, we analyzed the CD3 expression in whole retinas by Western blotting and immunohistochemistry. As shown in **Figure 2A**, the level of CD3 was increased significantly in 12-month-old h3T-A2 mice when compared to age-matched wild-type HLA-A2 mice (h3T-A2: 174±27% vs. HLA-A2: 100%; *p* = 0.04; n = 6). **Figure 2B** shows no staining for CD3 in the wild-type-HLA-A2 mice; however, the staining for CD3 was clearly increased in h3T-A2 transgenic mice. Strong immunostaining was observed for CD3 in the nerve fiber layer and retinal ganglion cell layer (shown by white arrow). The CD3 staining seen in outer plexiform layer indicated by arrowhead is non-specific as it was also seen in negative control when anti-CD3 antibodies were omitted. As noted in the h3T-A2 mice, the nuclei in the nerve fiber layer and RGC layers appear to be shrinking in size when compared with the HLA-A2 mice.

**Figure 3:**
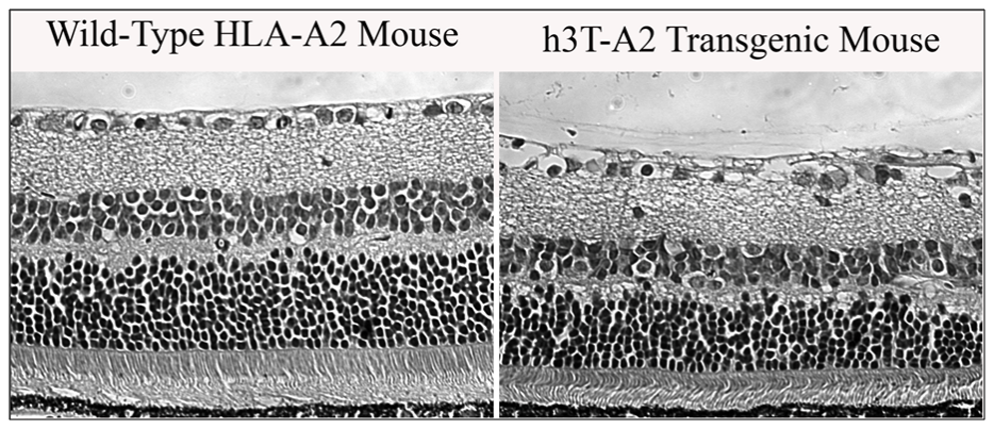
Morphological evidence of retina damage in h3T-A2 transgenic mice. Representative photomicrographs comparing morphologic changes in the 12-month-old wild-type HLA-A2 and h3T-A2 transgenic mice. Retina sections were stained with hematoxylin and eosin.

To determine the morphological deficits, we have performed hematoxylin and eosin staining and retina morphology was compared between 12-month-old h3T-A2 mice and wild-type HLA-A2 mice (**Fig. 3**). Overall retina thickness was significantly decreased by 24% (**Table 1**). This reduction in thickness was primarily due to significant thinning of the nerve fiber layer (NFL), inner plexiform layer (IPL), inner nuclear layer (INL), and outer nuclear layer (ONL) by 16%, 23%, 27%, and 30%, respectively, when compared to wild-type HLA-A2 mice (**Table 1**).

**Table 1:** Thickness of retina and individual layers measured in 12-month-old wild-type HLA-A2 and h3T-A2 transgenic mice.

	Retina (µm)	NFL (µm)	IPL (µm)	INL (µm)	OPL (µm)	ONL (µm)
HLA-A2	161.3±5.8	13.03±0.71	38.83±0.39	28.37±0.94	12.76±0.58	44.37±2.0
h3T-A2	122.0±2.0*	10.92±0.56*	30.37±2.13*	20.60±1.55*	10.68±0.70*	31.20±2.50*
*p*-Value	0.0001	0.048	0.002	0.0006	0.037	0.0001

Values are (mean ±SE), n = 3–4. Values were compared between groups using one-way ANOVA with Dunnett's post-test.

*indicates significant difference from wild-type eye (*p*<0.05). NFL, nerve fiber layer; IPL, inner plexiform layer; INL, inner nuclear layer; OPL, outer plexiform layer; ONL, outer nuclear layer.

### Interferon gamma (IFN-γ) mediated spontaneous vitiligo in h3T-A2 transgenic mice

**Figure 4:**
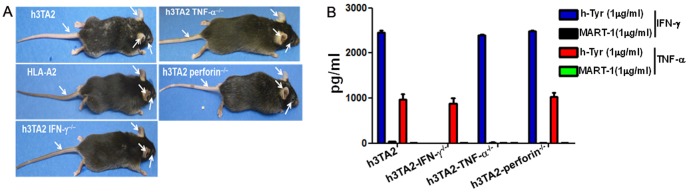
(**A**) Control mouse and h3T-A2 mice on deficient backgrounds as indicated. White arrows point to de-pigmentation of ear pinna and tails observed in these h3T-A2 pups at time of weaning (day 21). (**B**) Splenocytes (n = 4) from age matched (8 weeks) effector molecules competent (h3TA2) or deficient (h3T-A2IFN-γ^-/-^, h3T-A2TNF-α^-/-^ and h3TA2Perforin^-/-^) mice were co-cultured overnight with cognate antigen h-Tyr (1 µg/ml) or control peptide (MART-1 at 1 µg/ml) pulsed surrogate antigen presenting T2-A2 cells. Supernatant were collected and analyzed for cytokine release by ELISA as per manufacturer's protocol.

Since various effector cytokines and cytolytic molecules have been shown to contribute toward melanocyte destruction [24]–[27], we developed the h3T-A2 transgenic mouse with deficiency for either IFN-γ, TNF-α, or perforin to understand the role of these molecules in vitiligo development. Intriguingly, genetic ablation of TNF-α or perforin had minimal effect on the development of vitiligo (Fig. 4A, right panel). However, the absence of IFN-γ halted the development of vitiligo in h3T-A2 mice. No signs of vitiligo were noticed in h3T-A2 IFN-γ^-/-^ mice even at five months of age (Fig. 4A, lower left). Expectedly, the h3T-A2, h3T-A2-Perforin^-/-^ and h3T-A2-TNF-α^-/-^ splenocytes secreted high level of IFN-γ after stimulation with cognate human tyrsoinase antigen (Figure 4B), while no IFN-γ was detected in the supernatant obtained from the activated h3T-A2 IFN-γ^-/-^ knockout mice. However, the h3T-A2, h3T-A2-Perforin^-/-^ and h3T-A2-IFN-γ^-/-^ splenocytes secreted high level of TNF-α after stimulation with cognate human tyrsoinase antigen and no TNF-α was detected in the supernatant obtained from the activated h3T-A2-TNF-α^-/-^ knockout mice. This data suggests it is not TNF-α, but the ability of transgenic T cells to secrete IFN-γ that contributes to development of autoimmune vitiligo in h3TA2 mice. This led us to investigate if loss of IFN-γ in h3T-A2 mice also correlates to reduced ocular pathology, and if there is a role of IFN-γ in ocular autoimmunity.

**Figure 5:**
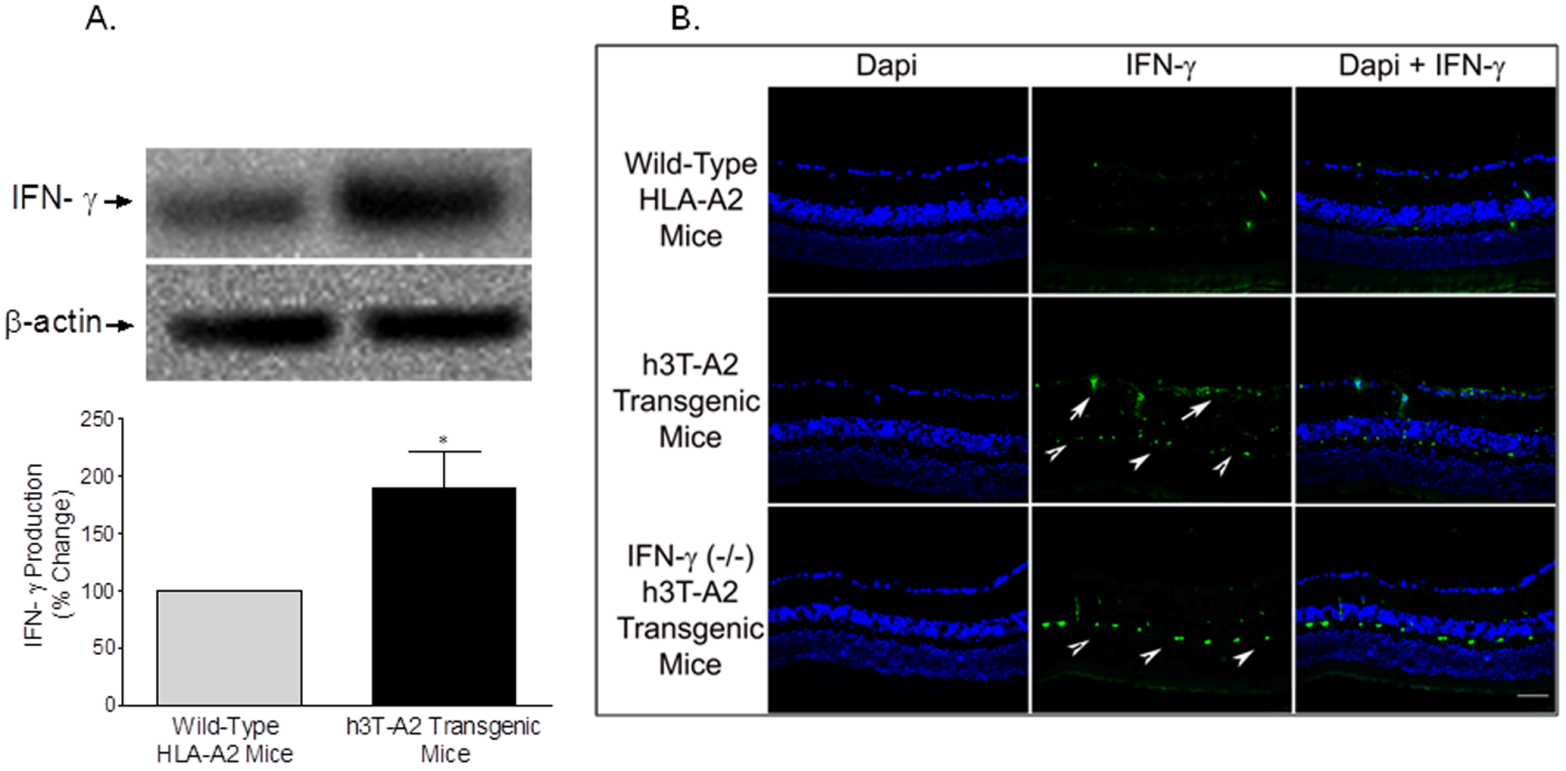
(**A**) Eyes of 12-month-old HLA-A2 and H3TA2 mice were enucleated and whole retinal extracts were analyzed by Western blotting using anti-IFN-γ antibodies. Band intensities of Western blots were quantitated by densitometry. Data are mean ±SE. *p<0.05; n = 11 for HLA-A2 and h3T-A2 groups. (**B)** Retinal cryosections of 12-month-old HLA-A2 and H3TA2 mice were immunostained by anti-IFN-γ (green) antibodies and nuclei were stained with DAPI (blue). There was no positive staining when primary antibodies were omitted (not shown). Bar size is 20 microns. Data is a representation of at least four independent experiments.

**Figure 6:**
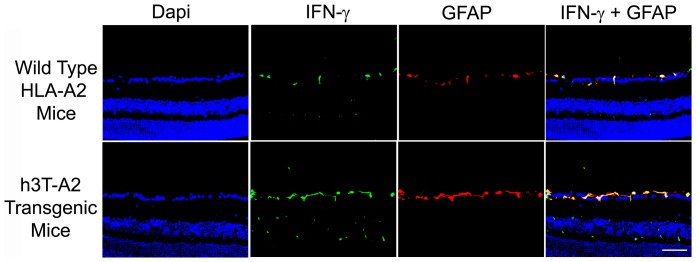
Eyes of 12-month-old wild-type HLA-A2 and h3T-A2 mice were enucleated and cryosections were immunostained by IFN-γ (green), anti-GFAP (red) antibodies, and blue for DAPI in the nuclei. There was no positive staining when primary antibodies were omitted (not shown). Fluorescence microscopy; bar size is 20 µm.

We further examined if the differences in IFN-γ secretion by CD3^+^ transgenic T cells could be responsible for retinal degeneration and RGC death in these strains of h3T-A2 mice. To test this, we determined the levels of IFN-γ in the retina of h3T-A2 mice. As shown in Figure 5A, the level of IFN-γ was increased significantly in 12-month-old h3T-A2 mice when compared to wild-type HLA-A2 mice (h3T-A2: 189±11% vs. HLA-A2: 100%; *p* = 0.023; n = 11). Figure 5B shows strong immunostaining for IFN-γ in the nerve fiber layer and retinal ganglion cell layer (indicated by arrows) with very mild staining in the inner plexiform layer. We have seen some non-specific staining in outer plexiform layer (indicated by arrowhead), which was also seen in negative control when anti-IFN-γ antibodies were omitted (data not shown). As expected IFN-γ staining was fully diminished in the IFN-γ^-/-^ h3T-A2 mice (Fig. 5B). Furthermore, we have performed a dual-immunolabeling using IFN-γ and GFAP (a glial cell marker). As shown in Figure 6, IFN-γ immunostaining was co-localized with GFAP immunostaining (far right panels), suggesting that IFN-γ is closely associated with glial cells. As noted in the h3T-A2 mice, the nuclei in the nerve fiber layer and RGC layers appear to be shrinking in size when compared with HALA-A2 mice.

### Pathological markers: Resemblance between h3T-A2 and ocular disease models

**Figure 7:**
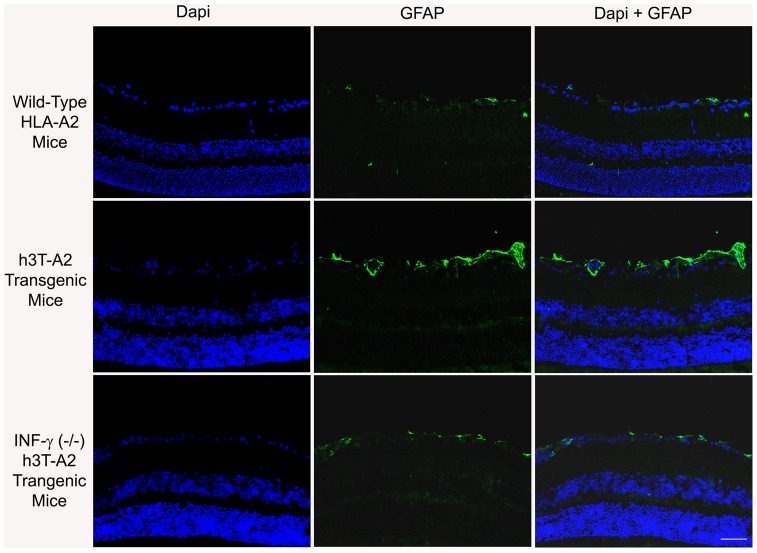
Eyes of 12-month-old wild-type HLA-A2 and h3T-A2 mice were enucleated and cryosections were immunostained by anti-GFAP antibodies. Green color indicates staining for GFAP and blue for DAPI in the nuclei. There was no positive staining when primary antibodies were omitted (not shown). Fluorescence microscopy; bar size is 20 µm.

**Figure 8:**
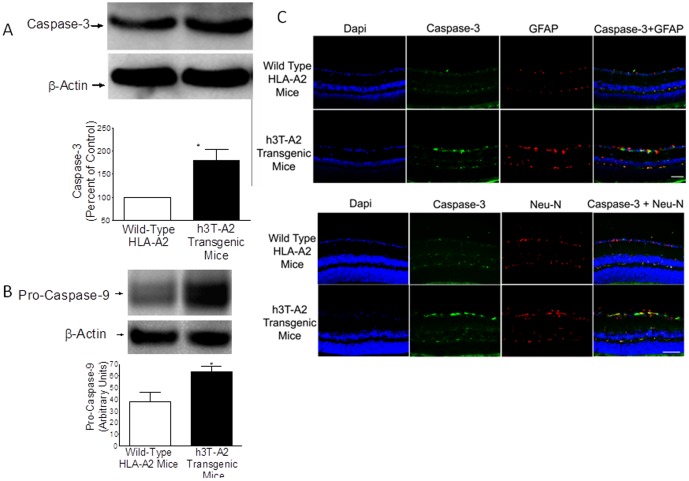
Eyes of 12-month-old HLA-A2 and h3T-A2 mice were enucleated and whole retina extracts were analyzed by Western blotting using anti-caspase-9 (A) or anti-caspase-3 (B) antibodies. Band intensities of Western blots were quantitated by densitometry. Data are mean ±SE, *p<0.05; n = 4–7 for HLA-A2 and h3T-A2 groups. (C) Retina cryosections of 12-month-old HLA-A2 and h3T-A2 mice were immunostained by anti-caspase-3 antibodies (green), anti-GFAP (red), and nuclei were stained with Dapi (blue). There was no positive staining when primary antibodies were omitted (not shown). Bar size is 20 µm. Data are a representation of at least four independent experiments.

Glial activation in response to glaucomatous injury in human glaucoma, and experimental glaucoma animal models has been demonstrated [28]–[31]. In addition to vascular abnormalities in diabetic retinopathy, glial activation (as measured by GFAP up-regulation) has been reported [32], [33]. To determine the impact of T cell/IFN-γ on glial activation in h3T-A2 mice, we stained retina sections with glial fibrillary acidic protein (GFAP, a glial cell marker) antibodies. An intense GFAP staining was seen in 12-month-old h3T-A2 mice, which was mostly localized to the nerve fiber layer. A punctate staining was also seen on the inner plexiform layer of the h3T-A2 mice when compared with age-matched HLA-A2 mice (**Fig. 7**). Interestingly, we have seen a remarkable reduction in GFAP staining in IFN-γ^-/-^ knockout h3T-A2 mice, suggesting that IFN-γ plays a key role in glial activation in h3T-A2 mice. To determine the downstream signaling events responsible for RGC death, we analyzed retinal samples for caspases (e.g., caspase-9 and -3) expression in h3T-A2 and HLA-A2 mice. As shown in **Figure 8A**, expression of pro-caspase-9 was up-regulated in h3T-A2 transgenic mice when compared with wild-type HLA-A2 mice (h3T-A2: 180±34% vs. HLA-A2: 100%; *p* = 0.043; n = 7). Additionally, we determined the expression of caspase-3, which has been shown to play a direct pathological role in RGC death. As shown in **Figure 8B**, expression of caspase-3 (activated form) was up-regulated in h3T-A2 transgenic mice when compared with wild-type HLA-A2 mice (h3T-A2: 179±24% vs. HLA-A2: 100%; *p* = 0.049; n = 4). Data in **Figure 8C** shows that caspase-3 staining was increased in retina layers of h3T-A2 mice, which was mostly localized to the inner retinal layers. A strong immunostaining for caspase-3 was seen in the nerve fiber layer and retinal ganglion cell layer with very mild staining in the inner plexiform layer. To identify the cell-type responsible for caspase-3 production, a dual-immunolabeling using GFAP (a glial cell marker) and NeuN (an neuronal marker) was performed. As shown in **Figure 8B**, caspase-3 immunostaining was more co-localized with NeuN than GFAP. However, some punctate overlapping immunostaining of caspase-3 and GFAP was also seen.

**Figure 9:**
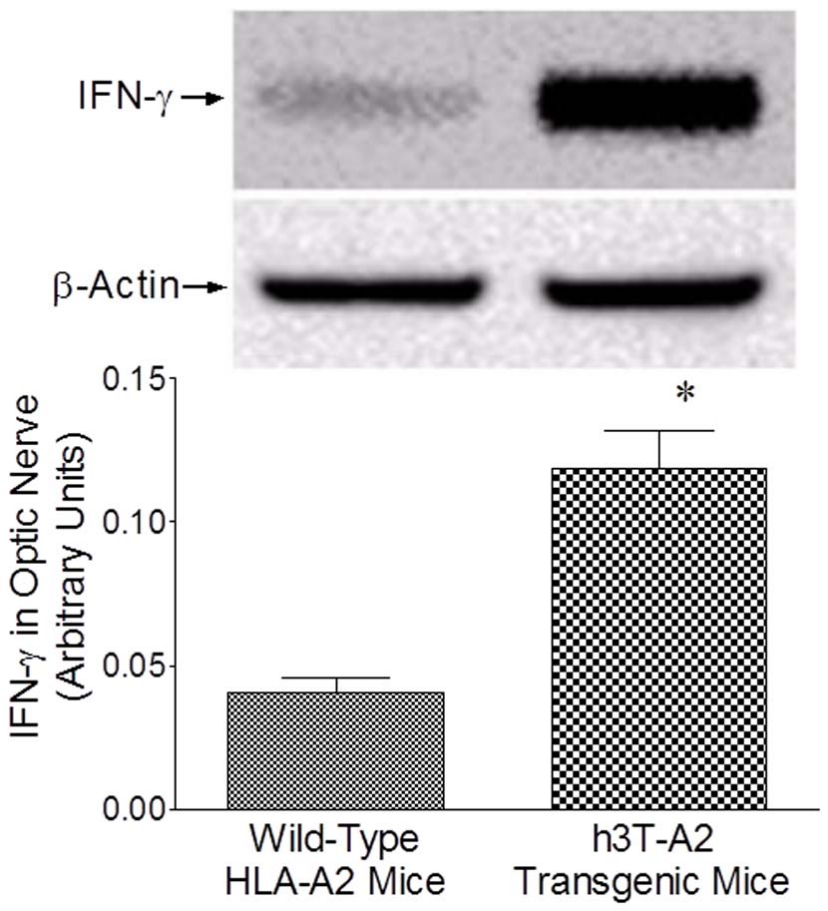
The optic nerves of 12-month-old HLA-A2 and h3T-A2 mice were removed and analyzed by Western blotting using anti-IFN-γ antibodies. Band intensities of Western blots were quantitated by densitometry. Data are mean ±SE, *p<0.05; n = 3.

We also have measured the changes in the expression pattern of IFN-γ in optic nerve of 12-month-old h3T-A2 and age-matched wild-type HLA-A2 mice. As shown in **Figure 9**, expression of IFN-γ was up-regulated in h3T-A2 transgenic mice when compared with wild-type HLA-A2 mice (h3T-A2: 295% vs. HLA-A2: 100%; *p* = 0.005; n = 3).

## Discussion

The precise role of the immune cells in retinal ganglion cell (RGC) death is not defined. Studies have shown that activation of T cells triggers the production of inflammatory cytokines and thereby initiates neurodegenerative responses. For example, increased levels of cytokines including interferon (IFN)- γ [34], interleukins (ILs) [8], [35], and tumor necrosis factor (TNF)- α [20], [36] have been reported to be associated with RGC death in numerous retinopathies including glaucoma. However, the precise role and the cellular events initiated by T cells and IFN-γ that facilitate RGC death during retinal injury remain largely unknown. To understand the pathological role of T cells and IFN-γ in retinal degeneration in general, and RGC death in particular, we utilized a unique T cell receptor (TCR) transgenic mouse model. This transgenic mouse was recently developed in our laboratory [18] using the HLA-A2 restricted high-affinity TCR reactive to human tyrosinase-derived peptide, YMDGTMSQV, isolated from class I-restricted CD4^+^ T cells from tumor infiltrating lymphocytes of a patient with metastatic melanoma [17]. In addition to depigmentation, the h3T-A2 mouse demonstrates visual defects such as loss of: retina function, RGC function, and RGC numbers. The ocular function begins to decline at the age of 9 months, which was significant at the age of 12 months in h3T-A2 transgenic mice when compared with age-matched HLA-A2 wild-type mice. In contrast, we have not observed any significant structural or functional changes in the retina of 6-month-old h3T-A2 transgenic mice (data not shown). In addition, the intraocular pressure (IOP) was not changed in the 6- to 12-month-old h3T-A2 transgenic mice when compared with HLA-A2 wild-type mice.

The progressive vitiligo observed in the h3T-A2 mice was due to melanocyte destruction [18]. Cytotoxicity of these tyrosinase-reactive and HLA-A2-restricted T cells was also confirmed by the loss of melanocytes from the hair follicles in depigmented mice as demonstrated by the loss of reactivity with antibodies to tyrosinase-related protein-1 (TRP-1). Furthermore, infiltration of the skin by antigen-specific, Vβ12 expressing T cells was also observed [18]. These results indicated that the expression of the TIL 1383I TCR on h3T and h3T-A2 T cells can lead to melanocyte destruction resulting in autoimmunity. Contrary to IFN-γ, the genetic ablation of TNF-α or perforin had no effect on the development of vitiligo, suggesting that the absence of IFN-γ led to complete control of the vitiligo in h3T-A2 mice.

Previous studies have shown that hypo-pigmentation due to mutation in the tyrosinase gene can cause deterioration in the retinal pigment epithelium (RPE) and photoreceptor function [37]. However, h3T-A2 transgenic mice were born pigmented since they retained the tyrosinase gene and the depigmentation seen in the h3T-A2 transgenic mice is due to melanocyte destruction by tyrosinase-reactive TCR. Having a reactive TCR within the eye, the most pigmented tissue being the RPE, could represent a potential target of tyrosinase TCR and may have resulted in the pathology in the RPE layer. To rule out this possibility, we performed optical coherence tomography (OCT) and H&E staining to monitor structural changes, and measured ERG a-wave amplitudes for photoreceptor response. We have not seen any apparent sign of pathology in the RPE as determined by OCT (data not shown) and H&E staining. However, more appropriate test for RPE function such as c-wave measurements will be needed to confirm RPE pathology, if any, in this h3T-A2 transgenic model. Additionally, we have seen a mild but insignificant decline in photoreceptor activity, as determined by a-wave amplitudes (data not shown). Overall, our results provide initial leads that the h3T-A2 mouse model exhibits degeneration of inner retinal layers and more pronouncedly affects the integrity and function of RGCs.

Our data also show that T cells infiltrate the inner retina, where production of IFN-γ was increased significantly in 12-month-old h3T-A2 mice when compared to wild-type HLA-A2 mice. Apart from a few studies that have shown that cytokines produced from both Th1 and Th2 cells are involved in RGC death in response to injury [38], there are no published data highlighting the direct pathological role of IFN-γ in RGC death. Thus, the increased infiltration of active transgenic T cells into the eye could result in retinal degeneration and RGC death/dysfunction. An earlier study used transgenic rats with constitutive expression of IFN-γ in the eye to study its paracrine effects and showed that chronic exposure of ocular cells to IFN-γ results in the apoptotic death of RGCs, development of chronic choroiditis, formation of retinal in-foldings, and activation of pro-inflammatory genes [39]. However, the role of IFN-γ in a normal physiological setting would require that key cells (T or NK) producing this cytokine to actually cross the blood-retina barrier and be present in an activated state to cause ocular dysfunction. Our recent study has shown that increasing the T cell tolerance by adoptively transferring regulatory T cells (Treg) in h3T-A2 mice blunts Th1 effector response and controls progression of vitiligo [40]. Similarly, it may be possible that an imbalance between Treg and effector T-cells in the h3T-A2 transgenic mouse, leads to increased infiltration of tyrosinase epitope reactive T-cells into the eye resulting in an increased accumulation of IFN-γ leading to RGC death. However, this needs to be determined in the future studies.

The data obtained from the h3T-A2 mouse model with reference to ocular dysfunction also seems to be partially in line with that observed in glaucoma. Glaucoma is a complex disease and numerous retina proteins have been demonstrated to be up-regulated during the pathogenesis of glaucoma, including TNF-α, TNF-R1, various protein kinases, glial cell activation, and proteolytic caspases [7], [20], [28]–[30], [41], [42]. Glial activation in response to glaucomatous injury in human glaucoma and experimental glaucoma animal models has been demonstrated. Similar to the glaucoma model, an intense GFAP staining was seen in 12-month-old h3T-A2 mice suggesting a sustained glial activation in the h3T-A2 mice, which may have subsequently directly/indirectly contributed in RGC death. This glial activation appears to be directly associated with IFN-γ-mediated pathways, because glial activation is markedly reduced in IFN-γ knockout h3T-A2 mice. Studies performed in experimental animal models of glaucoma and human glaucoma autopsy, suggest a key role for the immune system in mediating neuronal cell death in glaucoma irrespective of the level of intraocular pressure [43], [44]. Additionally, a potential pathogenic role for autoimmunity in facilitating neuronal cell loss in glaucomatous optic neuropathy [45] has been reported. The immunoregulatory mechanisms that determine RGC fate are influenced not only by elevated intraocular pressure (IOP), but numerous non-IOP dependent stressors including cytotoxic auto-antibodies, oxidative stress, and reactive oxygen species play critical roles in RGC death. Studies also have shown that RGCs die by apoptosis in rat, rabbit, and monkey glaucoma models [46], [47], and in human glaucoma [48]. There is a lack of clear evidence supporting T cell invasion in glaucoma; however, peripapillary chorioretinal atrophy zones, optic disc hemorrhage, and alterations in the perivascular barrier may facilitate the access of systemic immune components to the retina and optic nerve tissues in glaucoma. In addition, abnormal T cell subsets in peripheral blood from patients with normal pressure glaucoma or primary open-angle glaucoma have been reported [49]. Consistent with serum alterations, aqueous humor levels of antibodies in glaucoma patients have also been reported [50]. Considering these observations and the evidence, it is reasonable to speculate that T cells could have infiltrated to the retina, where they could facilitate RGC death. In diabetic retinopathy, studies also have shown an early RGC death [32], [51], [52], degeneration of retinal layers and function [51], [53], and glial activation [32]. Numerous clinical studies have indicated that Interferon (IFN)-treatment causes retinopathy in 15–64% of IFN-treated patients, transforming this complication into a significant risk for visual impairment [54]. Treatment with IFN causes hemorrhages, cotton wool spots, branch or central retinal artery occlusion, central retinal vein occlusion, anterior ischemic optic neuropathy, optic disc edema, neovascular glaucoma and vitreous hemorrhage. More specifically, clinical studies have shown a development of neovascular glaucoma in the course of IFN-α therapy for hepatitis C. The diabetic retinopathy in such patients has also worsened since the initiation of IFN-α therapy. Interestingly, such ocular symptoms were improved when IFN-α was discontinued [55], [56]. In other clinical study, IFN therapy to treat chronic hepatitis C leads to the development of central retinal vein and artery occlusion, retinal ischemia, and neovascular glaucoma [57]. In addition, clinical studies have shown increased levels of IFN-α and INF-γ in aqueous humor [58] and tears [59] of glaucoma patients. To begin to dissect-out early signaling targets in h3T-A2 transgenic mice, we have analyzed the changes in the expression of caspase-3 and caspase-9. Up-regulated caspases in h3T-A2 transgenic mice may have initiated the activation of downstream signaling cascades (e.g., activation of pro-apoptotic proteins), subsequently leading to RGC death. However, additional studies are needed to test these speculations and sequence of events that can lead to the RGC death.

In summary, it is evident that the h3T-A2 transgenic mice exhibit visual defects that are mostly associated with the inner retinal layers and RGC function, while the IOP remains unchanged in the h3T-A2 transgenic mice. In addition to the loss in retina function, T cell infiltration and IFN-γ expression were increased within the retina, which may have subsequently caused RGC death. This novel h3T-A2 transgenic mouse model provides an exceptional opportunity to understand RGC pathology without IOP manipulation and/or mechanical damage to the optic nerve. Furthermore, this model will be highly useful to understand the molecular mechanisms of RGC death in retinopathies in which RGCs are at high risk. Additionally, this model will provide a unique opportunity to institute neuroprotective strategies to protect RGCs.
